# Comparison of ultrasound with computed tomography to measure skeletal muscle mass in critically ill patients: A prospective study protocol

**DOI:** 10.1097/MD.0000000000031921

**Published:** 2022-12-02

**Authors:** Leandro Moreira Peres, Fabio Luis-Silva, Mayra Gonçalves Menegueti, Lucas Sato, Anibal Basile-Filho, Vivian Marques Miguel Suen, Olindo Assis Martins-Filho, Maria Auxiliadora-Martins

**Affiliations:** a Division of Intensive Care Medicine, Department of Surgery and Anatomy, Ribeirão Preto Medical School, University of São Paulo, Ribeirão Preto, Brazil; b Ribeirão Preto Nurse School, University of São Paulo, Ribeirão Preto, Brazil; c Department of Internal Medicine, Ribeirão Preto Medical School, University of São Paulo, Ribeirão Preto, Brazil; d René Rachou. Institute, Oswaldo Cruz Foundation, FIOCRUZ-Minas, Belo Horizonte, Brazil.

**Keywords:** computed tomography, critically ill patients, intensive care unit, muscle thickness, nutritional assessment, ultrasound

## Abstract

Therapy and nutritional status directly interfere in the clinical evolution of critically ill patients, in reducing morbidity and mortality, by maintaining the functional integrity of the gastrointestinal tract, decreasing the catabolic response, besides contributing to the reduction of hospitalization time resulting in less treatment cost. Critical patients and trauma victims suffer early changes in the quantity and quality of muscle mass. Tools to identify the groups most susceptible to these complications are necessary so that interventions can minimize the deleterious effects of malnutrition in critically ill patients.

**Methods and analysis:**

The aim of the present study is to measure muscle mass loss by measuring the thickness of the rectus femoris muscle by bedside ultrasound in critically ill patients admitted to the Intensive Care Unit (ICU) of a university hospital. Information will be collected regarding the length of hospital and ICU stay, the reason for admission, anthropometric data at admission and during hospitalization, energy needs, nutritional therapy used, and fasting time. This is a prospective, observational study that will be carried out in a single center in an ICU of a tertiary university hospital. The study population will undergo 3 tomographic images and 3 ultrasounds of the rectus femoris of each patient at different times. We propose, unprecedentedly, performing a validation study of ultrasound with the gold standard Computed tomography to evaluate the musculature of critically ill patients victims of traumatic brain injury. The results got will texto be fundamental for the development of new fields of investigation and certainly contribute to the discovery of a new approach to treat sarcopenia in critically ill patients. The Research Ethics Committee approved the study and all patients included will sign an informed consent form. (Clinical Record: RBR-2bzspnz).

## 1. Introduction

### 1.1. Background and rationale

Prolonged stays in intensive care units (ICU) are associated with sarcopenia, which is muscle atrophy associated with age and the progression of a disease.^[1]^ Its development has been related to the process of inflammation and immobilization,^[2]^ being a frequent finding in critically ill patients that may begin within the first 24 hours of admission.^[3]^ Acquired weakness in ICU patients affects 24% to 77% of patients hospitalized for over 1 week.^[4]^

The main risks for the development of muscle weakness are sepsis, mechanical ventilation, use of catecholamines, hyperglycemia, prolonged ICU stay and immobility.^[4,5]^

It is essential to use technologies for body mass assessment to identify sarcopenic patients on admission and document longitudinal changes in musculature during ICU stay.^[6]^

Body mass index (BMI) has been commonly used to identify critically ill patients at risk of malnutrition, but BMI does not distinguish between tissues such as muscle mass, fat, or edema during hospitalization^[6]^ Other markers, such as serum albumin, prealbumin, among others, do not reflect the nutritional reality of critically ill patients, since there is an increase in acute phase protein synthesis at the expense of albumin synthesis in cases of severe acute diseases^[7]^

Studies suggest that muscle wasting is an inevitable consequence in unconscious patients, and can reach 65% in 42 days in critically ill patients.^[8]^

Factors such as age, muscle function, medications, comorbidities, malnutrition, stress and muscle damage can contribute positively to the occurrence of damage and negatively to the ability to functional recovery.^[2]^ Mortality in sarcopenic patients is more than double of non-sarcopenic patients (32% vs 14%).^[9]^ There is also an association of sarcopenia with difficulty in weaning from ventilation, prolonged hospital stay, and prediction of mortality at admission within 1 year.^[3,4,6]^

It is challenging to identify patients who are at high risk for developing muscle weakness associated with ICU admission.^[10]^ Changes in muscle mass can induce dysglycemia and increase in inflammatory cytokines.^[11]^ Patients who survive ICU admission are left with sequelae and muscle weakness, and these conditions can persist for many years.^[2,3,12]^

Lower limb muscles are prone to early atrophy, shown by a greater decrease in thickness in the first few days of ICU admission compared to the upper limbs, which makes these muscles a good target for muscle mass assessment.^[12–14]^

The current techniques for assessing muscle thickness are: magnetic resonance imaging and computed tomography (CT), the latter considered the gold standard. However, these techniques have a high cost and the inconvenience of not being in the same environment as the critical patient.^[13]^ Other less expensive features, such as dual energy *X*-ray absorption, bio-impedance analysis are influenced by confounding factors such as excess fluid,^[15]^ and this situation is common in critically ill patients. Muscle measurement using ultrasound (USG), although it needs to be validated, is an easy-to-perform, accessible, safe, low-cost technique that does not use radiation and seems to be useful in the assessment of muscle mass to diagnose sarcopenia.^[2,10,12–14,16–20]^

### 1.2. Research hypothesis

The assessment of skeletal muscle mass through ultrasound agrees with the gold standard method CT.

## 2. Study objectives

### 2.1. Primary outcome

The primary endpoint will be muscle group (quadriceps) thickness. The agreement of quadriceps thickness will be verified between the images got through point-of-care ultrasound, a fast, low-cost, noninvasive method available in the ICU versus CT, the gold standard for assessing muscle thickness.

### 2.2. Secondary outcome

The secondary outcome will include nutrition of trauma patients, associating the loss of muscle mass with caloric and protein intake and laboratory changes caused by the stress of traumatic brain injury, such as levels of creatinine, albumin, total protein fractions, protein c reactive, blood count, platelet count and bilirubin.

### 2.3. Design

Prospective, observational study to validate the use of US to measure muscle thickness in ICU patients. The research will be carried out in an ICU of a tertiary university hospital.

## 3. Patients and methods

### 3.1. Study participants and eligibility criteria

Patients over 18 years of age, admitted to the ICU, victims of traumatic brain injury, will be included. During the acquisition of CT scans of the skull, a CT scan of the quadriceps will be performed to assess the thickness of the muscle group and compared to USG. At each CT of the control skull, at the following times: 24 hours to 96 hours and 96 hours to 168 hours after admission, controls on the thickness of the quadriceps will be performed using CT and USG and we will compare these measurements for validation of the method.

Patients with any known neuromuscular disease, such as myopathy, neuropathy or stroke, any lower limb amputated, who have undergone lower limb orthopedic surgery, or with fractures, injuries or burns in the region to be studied, pregnant women, metastatic cancer, in end-of-life care, requiring home ventilation, in prone position, transferred from another hospital after hospitalization for over 48 hours and patients in state custody. All selected patients and/or legal guardians will be advised about the study and if they agree to take part, they will sign the informed consent form (ICF).

### 3.2. Randomization and allocation

There will be no randomization, as all patients will undergo the exams. After the study team confirms that a subject meets all the study entry criteria, they will be included and all patients will receive a copy of the informed consent form to take part in the study, besides the verbal explanation of the step-by-step of the study.

### 3.3. Study procedures

#### 3.3..1. *Study protocol*.

Anthropometric, clinical and nutritional data will be collected: height, weight, BMI, injury severity score,^[21]^ prognostic scores SAPS-3^[22,23]^ and APACHE II,^[24]^ number of days of mechanical ventilation and ICU and hospital stay,^[3]^ NUTRIC score,^[25]^ NRS 2002^[26]^ and basal energy expenditure through pocket formula with calorie and protein requirements.

Weight will be measured on a Stryker scale stretcher, height will be used standard body tape measure, measurements will be taken at the time of admission of the patient to the ICU. BMI will be classified according to the World Health Organization 1995.^[27]^

Daily, the volume of diet and protein intake will be recorded until the seventh day of hospitalization, and the percentage of diet intake will then be calculated by the ratio of the amount actually ingested to the amount prescribed.

**Figure 1.**
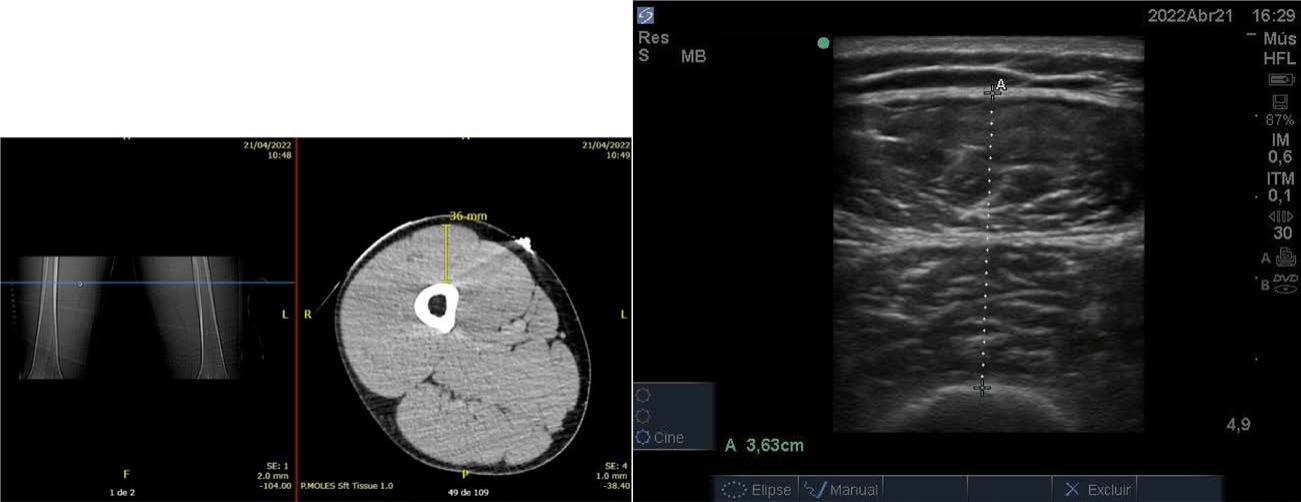
Illustrative photo to characterize the gained images (CT and USG). CT = computed tomography, USG = ultrasonography.

The USG will be performed with a Sono Site M-Turbo device with 2-dimensional mode, with a linear transducer (frequency: 7–13MHz) to get high-resolution images of surface structures 1, in cross-section, in B-mode, with the transducer oriented transversely to the longitudinal axis (the imaginary line marked before) of the thigh forming a 90° angle in relation to the skin surface with minimal pressure on the skin (Fig. 1).

The measurement will be performed with the patient in a supine position with the leg extended and relaxed, at the midpoint between the anterosuperior iliac spine and the proximal edge of the patella, where the quadriceps femoris will be visualized, a muscle group in the thigh, formed by 4 muscles: vastus medialis, intermedius, lateral and rectus femoris.^[15]^ Subsequently, we will use planimetric techniques to measure distances and areas: Thickness: from the muscle-fat interface to the muscle-muscle interface or from the muscle-fat interface to the bone surface,^[8]^ forming a straight horizontal line and; manually describing the thickness of the quadriceps femoris.

**Figure 2.**
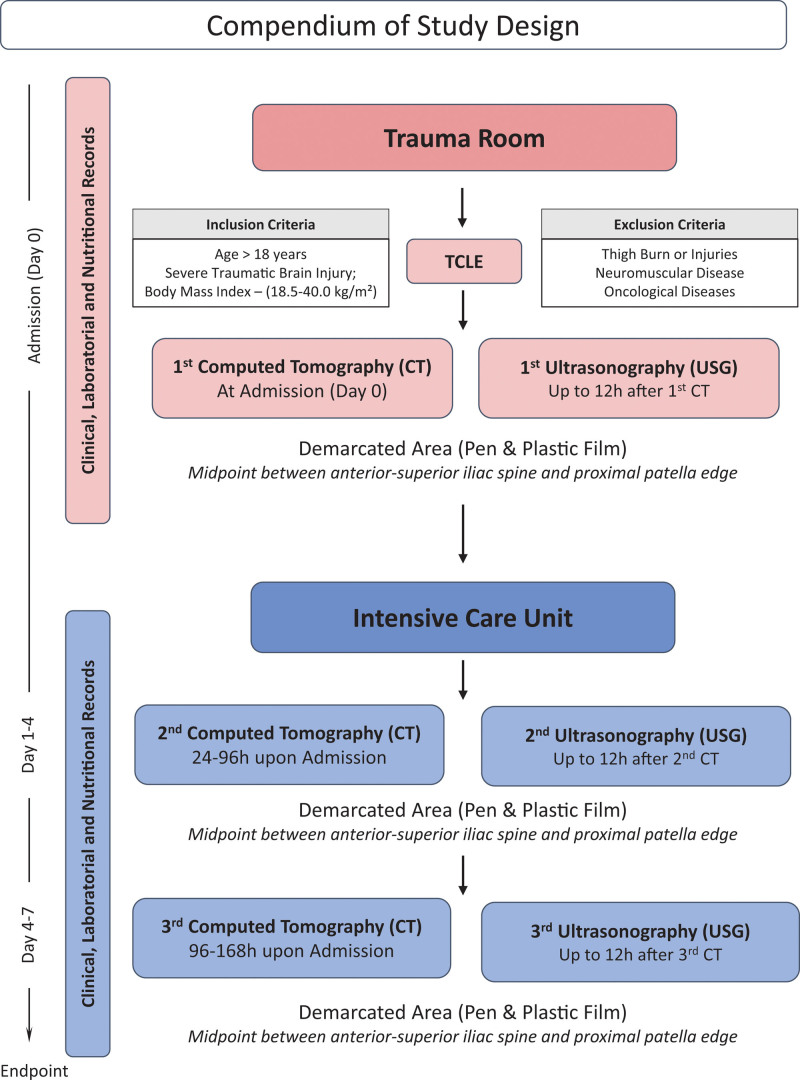
Compendium of study design.

Measurements will be got at the time of admission of the patient to the hospital, preferably still in the trauma room, within 24 hours of the trauma. Second measurement 24 hours to 96 hours after admission, preferably in the ICU and third measurement 96 hours to 168 hours after admission. All USG measurements will be performed simultaneously with quadriceps CT, with a maximum interval of 12 hours between 1 technique and another. The study protocol is represented in Figure 2.

Measurements will be got by 2 operators, intensive care physicians, trained in using bedside USG in the ICU. The standardization of USG measurements was previously published, whose correlation analysis found an excellent interobserver relationship (*R*2 > 0.90)^[28]^

Care will be taken during the handling of patients so that all measurements do not entail greater risks for them, except for the discomfort caused by the exam. The same examiner will make all measurements and, at the same point, as we will mark the studied limb with a marker pen and cover with plastic film.

We will perform the tomographic control exams of the thigh only in the studied region through the marking performed, to reduce: radiation dose to patients, costs and exam time. The CT analyzes will be interpreted by a radiologist and we will have 3 images per patient.

### 3.4. Statistical analysis

Initially, the Shapiro–Wilk test will be performed to evaluate the data distribution. Mean or median depending on the distribution of variables will describe data. To compare the values of quadriceps femoris thickness, Student’s t test or Wilcoxon test for paired samples will be applied, depending on the distribution of variables. Bland & Altman test will also be performed to assess the agreement between the methods. The relationship between caloric and protein intake, laboratory alterations and loss of muscle mass will be evaluated through multiple linear regression, with muscle mass being considered a dependent variable and caloric and protein offers and laboratory alterations being independent variables. We will use the STATA version 14 program for the analyses.

### 3.5. Data management

Data will be collected directly from the USG and CT device and will be stored to ensure reliability.

All participants will receive a numerical identification of the study itself. Personal data will be stored in the hospital’s digital system, protected by a password to ensure confidentiality.

Any modifications or side effects to the protocol will be communicated to REBEC.gov and to the Research Ethics Committee and clinical research unit of Hospital das Clínicas da Faculdade de Medicina de Ribeirão Preto by the responsible researcher.

All principal investigators will have access to the final trial dataset.

## 4. Discussion

Skeletal muscle function depends not only on the quantity of muscle but on the quality, and this is also adversely affected in critically ill patients. Changes in muscle echogenicity that occur early in critically ill patients are related to necrosis and myofibrosis when evaluated in histological sections.^[29]^

In assessing strength, the medical research council sum score < 48/60 has been used, a subjective analysis tool that is not able to accurately assess. Muscle biopsy and neural conduction tests are more accurate measures, but they are more invasive and less feasible in clinical practice.^[10]^

Recent studies show that the echogenicity of muscle ultrasound is correlated with muscle architecture, fat infiltration, fibrosis, and muscle necrosis. Normal musculature is hypoechogenic (dark). An increase in echogenicity, a lighter or brighter appearance, may represent muscle necrosis or fatty infiltration.^[10]^ The early identification of these patients is important, because they are predictors of functionality, morbidity and mortality, and their early identification can change their clinical outcome.^[10]^

Although some studies,^[30,31]^ using USG have shown disappointing results, USG performed strictly none of them in critically ill patients on mechanical ventilation and used the sectional area as a method of measuring muscle loss. Evaluation of quadriceps muscle thickness by USG revealed over the first week of critical illness a 5% greater chance of mortality at 60 days.^[32]^

A published study,^[33]^ used ultrasound to recognize muscle wasting in patients with sepsis in the ICU, found a significant association between muscle loss, infection and organ dysfunction, besides patients who had greater muscle mass loss having more complications after ICU discharge and greater mortality rate. It was identified that the most significant losses of muscle mass occurred in the first days of hospitalization and preventive care in this muscle loss should be performed as soon as possible. Thus, the authors recommend the use of ultrasound as a tool to predict prognosis in patients with sepsis in the ICU.

Decreased muscle thickness in mechanically ventilated patients showed that quadriceps muscle layer thickness loss was associated with 60-days mortality.^[32]^

Another observational study,^[34]^ aimed to evaluate the role of muscle ultrasound in the analysis of nutritional status in 29 patients admitted to the ICU, identified the greatest loss of muscle mass in the first week of hospitalization, having a direct association with the patient’s nutritional status. Although BMI is a commonly used parameter for screening nutritional status, it does not show the amount of muscle loss in these patients.^[35,36]^ In contrast, USG is a valuable and practical measurement tool that may hold promise in helping to guide metabolic support that allows morphological changes in muscles to be assessed in critically ill patients.^[37]^

It is also important to note how safe and viable the tool is. In patients with severe trauma, the use of quadriceps USG was easy to perform and reliable even in the hands of less experienced operators.^[38]^ Thus, USG ends up gaining ground for its ease of use and lower costs.

Our study can contribute to the use of USG in critically ill patients with muscle loss, not only by recognizing patients with a more fragile nutritional component but also by differentiating the more severe patients who are at greater risk of unfavorable evolution in the ICU. We believe USG is a noninvasive and easy-to-operate tool to distinguish patients on mechanical ventilation who have muscle wasting by analyzing quadriceps thickness, and thus early approaches could be applied to optimize treatment.

The novelty and importance of this project lies in using a method for assessing muscle mass through ultrasound of the quadriceps femoris, a noninvasive, low-cost bedside method that is feasible in critically ill patients.

In this study, we propose, unprecedentedly, performing a validation study of ultrasound with the gold standard (GS) to evaluate the musculature of critically ill patients victims of traumatic brain injury. The results got will be fundamental for the development of new fields of investigation and may contribute to the discovery of a new approach to treat sarcopenia in critically ill patients.

This study has limitations, such as the characterization of the study population in terms of age and sex, trauma severity, fluid balance, and associated comorbidities, which can affect both muscle thickness and outcomes. In addition, this is an observational study in a single center, which certainly has limitations in the patient profile and regional variations.

## Author contributions

**Conceptualization:** Anibal Basile-Filho.

**Data curation:** Leandro Moreira Peres, Olindo Assis Martins-Filho.

**Funding acquisition:** Maria Auxiliadora-Martins.

**Investigation:** Leandro Moreira Peres.

**Methodology:** Mayra Gonçalves Menegueti, Olindo Assis Martins-Filho.

**Project administration:** Maria Auxiliadora-Martins.

**Software:** Lucas Sato.

**Supervision:** Anibal Basile-Filho, Vivian Marques Miguel Suen, Maria Auxiliadora-Martins.

**Validation:** Vivian Marques Miguel Suen, Maria Auxiliadora-Martins.

**Visualization:** Olindo Assis Martins-Filho.

**Writing – original draft:** Leandro Moreira Peres, Fabio Luis-Silva, Maria Auxiliadora-Martins.

**Writing – review & editing:** Mayra Gonçalves Menegueti, Olindo Assis Martins-Filho, Maria Auxiliadora-Martins.
